# Predicting Intramyocardial Hemorrhage Before Reperfusion in STEMI Patients With Intrinsically Explainable Artificial Intelligence

**DOI:** 10.1016/j.jacadv.2026.102864

**Published:** 2026-06-11

**Authors:** Khalid Youssef, Keyur P. Vora, Rajesh Gupta, Gabriel Gruionu, Andreas Kumar, Rishi Puri, Grant W. Reed, Ankur Kalra, Rohan Dharmakumar

**Affiliations:** a Cardiovascular Imaging Research Center, Medical Imaging Research Institute, Indiana University School of Medicine, Indianapolis, Indiana, USA; b Krannert Cardiovascular Research Center, Indiana University School of Medicine, Indianapolis, Indiana, USA; c Division of Cardiology, Department of Medicine, University of Toledo, Toledo, Ohio, USA; d Division of Cardiology, Department of Medicine, Northern Ontario School of Medicine University, Sudbury, Ontario, Canada; e Division of Cardiovascular Medicine, Heart, Vascular and Thoracic Institute, Cleveland Clinic, Cleveland, Ohio, USA; f Division of Cardiology, Department of Medicine, State University of New York (SUNY), Upstate Medical University, Syracuse, New York, USA

**Keywords:** cardiac magnetic resonance, explainable artificial intelligence, intramyocardial hemorrhage, percutaneous coronary intervention, risk stratification, ST-segment elevation myocardial infarction

## Abstract

**Background:**

Intramyocardial hemorrhage (IMH) complicates approximately 40% of reperfused ST-segment elevation myocardial infarctions (STEMIs) and is associated with worse outcomes. No method identifies patients at risk before reperfusion.

**Objectives:**

The objective of the study was to develop and evaluate an explainable artificial intelligence approach for pre-reperfusion IMH prediction and translate it into a practical bedside score.

**Methods:**

We enrolled 288 STEMI patients from the MIRON-PREDICT clinical study (NCT06423625) between June 2023 and December 2024, of whom 252 were used for model development and 36 were used for validation. Electrocardiographic, angiographic, and clinical variables were obtained during emergency coronary angiography. Cardiac magnetic resonance imaging 48 to 72 hours post–percutaneous coronary intervention served as the reference for IMH. A Superposable Neural Network identified key predictors and informed derivation of a point-based scoring model for bedside use.

**Results:**

The final 3-variable model comprised the presence of coronary collaterals, degree of coronary artery occlusion, and sum ST-score. Cardiac magnetic resonance imaging identified IMH in 142/288 patients. Coronary collaterals were present in 144/288 and total occlusion in 131/288. Sum ST-score was substantially higher in IMH-positive than IMH-negative patients. The resulting model achieved 84.9% accuracy, 82.3% sensitivity, and 87.3% specificity in identifying patients at risk of IMH before reperfusion. This was confirmed in the validation cohort with 83.3% accuracy, 81.3% sensitivity, and 85% specificity.

**Conclusions:**

Explainable artificial intelligence enabled rapid integration of clinical parameters from cardiac catheterization procedures to accurately predict IMH in STEMI before revascularization. The proposed approach could guide clinical decisions in real-time. This could inform future strategies aimed at reducing the incidence of IMH.

Myocardial infarction (MI) is a significant global health concern, with over 800,000 cases reported annually in the United States alone.^1^ It is characterized by a sudden disruption of coronary blood flow, leading to ischemic injury to the myocardium. Patients experiencing ST-segment elevation MI (STEMI) require immediate medical intervention to minimize myocardial damage and improve survival rates.^2^ Despite advances in treatment methods, including percutaneous coronary intervention (PCI) reperfusion therapy, patients recovering from STEMI face considerable risks. One of the most concerning complications associated with reperfusion therapy is intramyocardial hemorrhage (IMH). IMH results from microvascular rupture following PCI and has been identified as the most severe form of tissue injury in reperfused STEMIs^3^^,^^4^ and is evident in approximately 40% of STEMI patients after PCI.^3^ Importantly, IMH is known to significantly exacerbate myocardial damage, abolish myocardial salvage following PCI, and increases the likelihood of major adverse cardiovascular events (MACE), including heart failure, fatal arrhythmias, and death.^5^, ^6^, ^7^, ^8^ Beyond its association with long-term MACE, recent multicenter evidence shows that hemorrhagic MI patients carry a ∼3-fold higher in-hospital mortality compared to those with nonhemorrhagic MI.^9^ These data underscore the clinical importance of IMH, heightening the imperative to identify patients at risk as early as possible.

T2∗ cardiac magnetic resonance (CMR) imaging is the gold standard for detecting hemorrhagic MI.^10^^,^^11^ Notably, T2∗-based diagnosis of hemorrhagic MI is performed days after reperfusion therapy; hence, it is retrospective in that it identifies hemorrhagic MI patients only after the development of IMH. At present, there is no method to prospectively identify STEMI patients at risk for hemorrhagic MI. If such an approach were to be available and one that can be exercised in the cardiac catheterization laboratory setting immediately before revascularization, it may open opportunities to intervene to mitigate the development of IMH. Finally, early risk stratification provides a rational framework for clinical trial design by enriching study populations for IMH, aligning endpoints with mechanistic targets, and increasing power to detect treatment effects of novel therapies.

To address this unmet clinical need, we developed a method to prospectively identify STEMI patients at the greatest risk of developing IMH using a fully explainable artificial intelligence (XAI). Although traditional bedside markers (eg, infarct territory, culprit-artery identity, and ischemia time) have shown variable associations with IMH across prior studies,^4^ and traditional multifeature "black-box" predictive models are difficult to interpret or validate in clinical settings, the proposed approach uses clinical data to develop a transparent, human-readable composite score to identify patients who are at risk of IMH at the time of emergency coronary angiography before revascularization (ie, intraprocedural, after diagnostic angiography in the catheterization laboratory).

## Methods

### Study population

**Figure 1:**
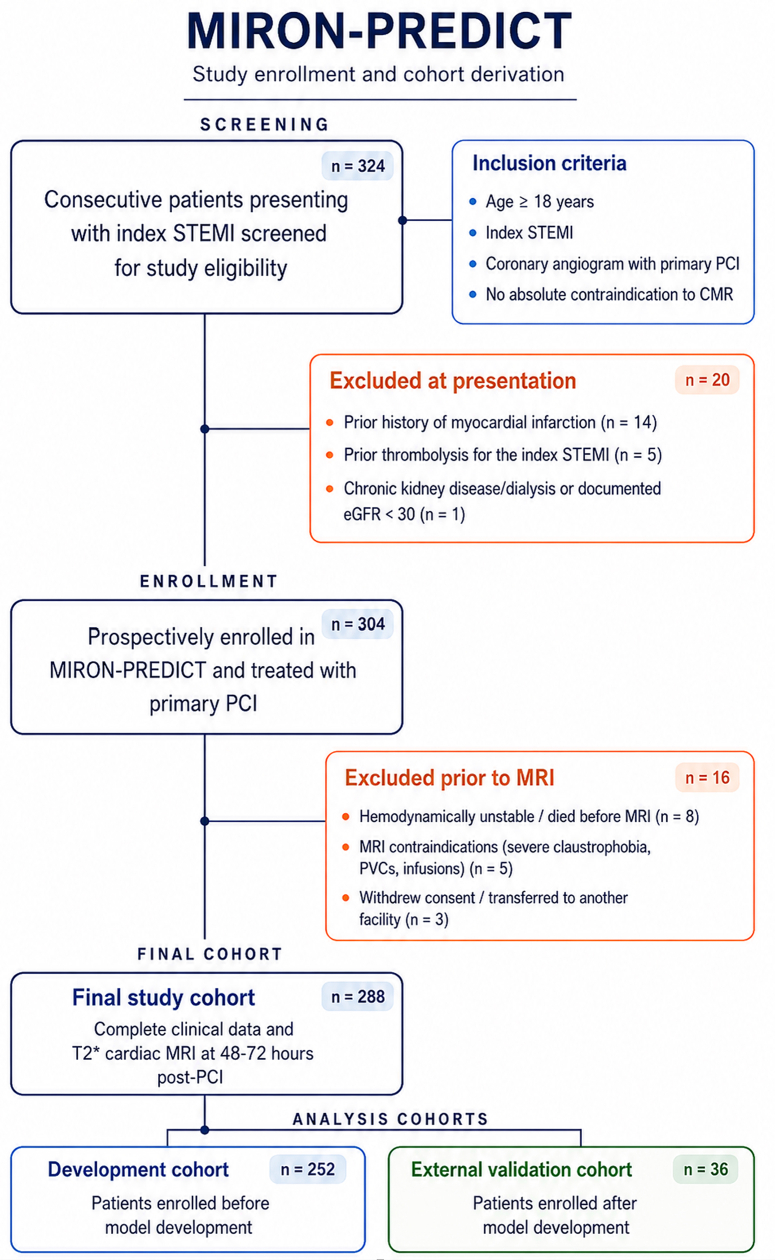
**Study Flow for the MIRON-PREDICT Cohort** Consecutive patients presenting with index ST-segment elevation myocardial infarction (STEMI) were screened for eligibility (n = 324). After exclusion at presentation (n = 20), 304 patients were prospectively enrolled and treated with primary percutaneous coronary intervention (PCI). A further 16 patients were excluded before cardiac magnetic resonance imaging (MRI), yielding a final study cohort of 288 patients with complete clinical data and T2∗ CMR imaging performed 48 to 72 hours after PCI. The final cohort was divided into a development cohort (n = 252; patients enrolled before model development) and an external validation cohort (n = 36; patients enrolled after model development). CMR = cardiac magnetic resonance; eGFR = estimated glomerular filtration rate; PVCs = premature ventricular contractions.

This study is based on the MIRON-PREDICT clinical study (NCT06423625), performed with the approval of institutional review board, included a cohort of STEMI patients (n = 252) experiencing their first event who underwent guideline-directed primary PCI. Patients were prospectively enrolled based on the availability of complete clinical data, including pre-PCI electrocardiogram (ECG), coronary angiography, and post-PCI CMR imaging data with T2∗ mapping. Post-PCI CMR imaging with T2∗ mapping was used to ascertain the presence of IMH. Inclusion criteria included patients >18 years of age and presentation with an index MI treated with primary PCI. Exclusion criteria included history of prior MI, thrombolysis before PCI, or contraindication for CMR imaging (Figure 1). An independent external cohort (n = 36) was used solely to prospectively validate the finalized point-based score and was not used for feature selection, model training, or threshold derivation.

### Data collection

Clinical data were collected during emergency coronary angiography and included a range of predictors that included catheterization laboratory (cath lab) and standard clinical (clinical) parameters. Angiographic predictors were taken from the diagnostic angiogram obtained before guidewire crossing and balloon inflation (ie, before restoration of flow). A total of 12 predictors were included in the study. Cath lab predictors included: extent of electrical abnormality determined based on sum of ST-segment elevation score; anatomical features of coronary obstruction (degree of coronary artery occlusion, presence of coronary collaterals, culprit artery [left anterior descending (LAD), left circumflex (LCX), or right coronary artery (RCA)], and lesion location [proximal, mid, or distal]). Clinical predictors included age, sex, body mass index, hypertension, diabetes, smoking status, and dyslipidemia. CMR imaging performed at 48 to 72 hours post-PCI was used as the gold standard to ascertain the presence of IMH. The final analytic cohort had complete data for the variables required by the score, including admission ECG-derived sum ST-score, pre-reperfusion culprit-vessel occlusion status, pre-reperfusion collateral status, and post-PCI T2∗ CMR outcome ascertainment. Therefore, no statistical imputation was performed.

### Sum ST-score – extent of electrical abnormality

The degree of ST-segment elevation has been shown to be a real-time indicator of microvascular injury.^12^ It was first proposed in 2010 as the best predictor of microvascular obstruction in patients treated successfully by primary PCI. A standard 12-lead ECG was recorded on admission at a paper speed of 25 mm/s and an amplification of 10 mm/mV. ECG data were subsequently evaluated by 2 cardiologists in consensus but blinded to patient identity, angiographic data, and CMR imaging findings. The isoelectric line was defined at the level of the preceding TP segment. Extent of ST-segment elevation was measured 20 ms after the J-point in every lead. Sum of ST-segment elevation was manually calculated as the sum of ST-segment elevation in all leads. It was measured immediately on admission, as this represents the earliest diagnosis of STEMI before any treatment in emergency room or intervention, ensuring an unbiased assessment prior to any influence of medications.

### Coronary angiography: lesion location, extent of occlusion, and collateral status

Coronary angiograms were performed immediately on arrival in the catheterization laboratory using a standardized emergency STEMI protocol via radial or femoral access. Nonionic contrast was injected under fluoroscopic guidance, and images were acquired using standardized cine angiographic views, including right anterior oblique/left anterior oblique cranial and caudal projections for optimal visualization of the culprit artery, lesion severity, and collateral circulation. Digital cine angiography was recorded at 15 frames per second, ensuring high-resolution imaging for assessment of TIMI flow and collateral grading. All angiograms were independently reviewed by 2 cardiologists, with consensus for all parameters. The culprit coronary artery was identified and categorized as the LAD, LCX, or RCA. Lesion location within the culprit artery was classified as proximal, mid, or distal. The degree of occlusion was also evaluated, with lesions categorized as either total (100% luminal narrowing) or subtotal (< 100% luminal narrowing) occlusion. Coronary collateral status was determined from pre-PCI coronary angiograms based on the Rentrop grading system:^13^ absent (no visible collaterals) or present (filling of side branches, partial filling of the epicardial coronary artery, or complete filling of the epicardial coronary artery). This grading reflects the presence of collateral circulation to the network of blood vessels supporting blood flow to the myocardial regions subtended by culprit artery during STEMI, which can influence both myocardial salvage and the risk of IMH.^14^^,^^15^

### CMR imaging – evidence of intramyocardial hemorrhage before patient discharge

To identify the presence of IMH, all patients underwent CMR imaging with T2∗ mapping within 48 to 72 hours post-PCI. The T2∗ mapping technique allows for the quantification of magnetic field inhomogeneities related to the presence of blood products such as iron deposits, which are strong indicators of IMH. CMR imaging was performed at 1.5-T (MAGNETOM Sola, Siemens Healthcare). T2∗ maps were constructed from multigradient-echo images acquired using repetition time (TR) = 15 ms, 8 echoes with echo times (TEs) = 3.2 ms to 13.7 ms with ΔTE = 1.5 ms, flip angle 15°, and bandwidth 810 Hz/pixel. Late-gadolinium enhancement images were acquired using segmented Phase-Sensitive-Inversion-Recovery reconstruction (TR/TE = 5.3 ms/2.0 ms, TI = 300 ms, flip angle 15°, and bandwidth = 355 Hz/pixel). MI regions with mean T2∗ values below 2 SDs of the remote myocardium were identified to be positive for IMH,^16^ with the volume of hemorrhage expressed as a percentage of the total left ventricular (LV) mass. To minimize false positives, similar to prior studies, only patients with IMH volume >4% of LV were considered to have had hemorrhagic MI.^3^ These findings were used as the ground truth to determine the incidence of IMH and its association with the collected clinical data.

### XAI methodology

**Figure 2:**
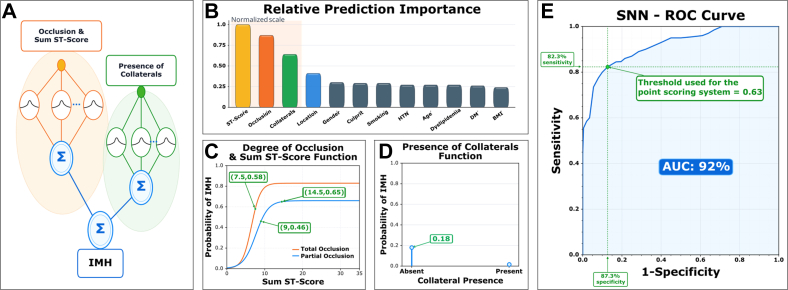
**Superposable Neural Networks Model Architecture and Results** (A) Superposable Neural Network (SNN) architecture. The deployed inference model is a compact additive network; training complexity reflects the optimization procedure, including teacher-student distillation, rather than the final model. (B) Relative importance of 12 candidate predictors. Coronary collaterals and the interaction between summed ST-segment elevation and degree of occlusion were sufficient for the final model. (C, D) Learned contribution functions showing how these predictors relate to intramyocardial hemorrhage (IMH) risk. (E) ROC analysis of the SNN. The operating threshold was set at 0.63, corresponding to the point closest to (0,1). Decision rules extracted from the additive functions were then translated into the point-based bedside score shown in Figure 3. AUC = area under the receiver operating characteristic; BMI = body mass index; DM = diabetes mellitus; HTN = hypertension; ROC = receiver operating characteristic.

We used the Superposable Neural Networks (SNNs) XAI approach to develop the predictive scoring model.^17^ The SNN method uses multiple stages of knowledge distillation as an optimization strategy to fit a highly constrained additive neural network architecture (student model) using soft targets produced by a high-capacity teacher model. Importantly, the teacher model is used only during training and is not used for inference or interpretation; intrinsic explainability comes from the final SNN itself, a special type of generalized additive model architecture in which each predictor (and explicit composite-feature interaction) contributes through an independent single-input function whose contributions sum exactly to the predicted risk. The SNN method was selected for its ability to provide both high predictive accuracy and intrinsic, full interpretability, such that a direct understanding of the clinical factors driving the development of IMH is possible. Two state-of-the-art models were considered for the teacher model and evaluated using 10-fold cross-validation, with area under the receiver operating characteristic (ROC) AUC values of 0.91 ± 0.03 (Random Forest) and 0.92 ± 0.03 (Multistage Training). Multistage Training was chosen as the highest performer. Unlike standard post hoc XAI methods (eg, Local Interpretable Model-agnostic Explanations and SHapley Additive exPlanations), which attempt to interpret already-trained black-box models via feature attribution,^18^, ^19^, ^20^, ^21^, ^22^, ^23^, ^24^ the SNN approach is inherently transparent by design. Interpretability is built into the model architecture itself, rather than being inferred after the fact. This is achieved through an additive deep learning architecture (Figure 2A) in which predictions are generated by summing independent functions, each representing a clinical predictor or a predictor interaction. The contribution of each component is explicitly presented, enabling end users to trace exactly how the final risk prediction is made.

Our XAI method also incorporates a robust feature selection stage, employing a tournament ranking system to evaluate all relevant clinical predictors and identify the most effective subset of *cath lab* and *clinical* parameters for IMH prediction. In addition to individual predictors, composite predictors (representing interactions between 2 or more features) were automatically generated and evaluated as independent variables. These composite predictors are essential for capturing the synergistic effects between variables, such as the interaction between ST-segment elevation and the extent of coronary artery occlusion. Noncontributing and redundant predictors were automatically discarded, leaving only the most relevant features and interactions. This refined subset was then used to train the SNN model, ensuring that every component of the final model contributes meaningfully and interpretably to the prediction outcome. In comparison, although logistic regression and decision trees can also be interpretable, they typically require either explicit a priori specification of nonlinear transformations and interaction structure (logistic regression) or can become unstable/complex when representing nonlinear risk functions (decision trees). In contrast, the SNN provides a deliberately constrained additive neural architecture that learns nonlinear single-input contribution functions (and explicitly selected composite-feature interactions) while preserving an exact per-feature decomposition of every prediction.

### Development of point-based predictive scoring model for rapid prediction of intramyocardial hemorrhage

**Figure 3:**
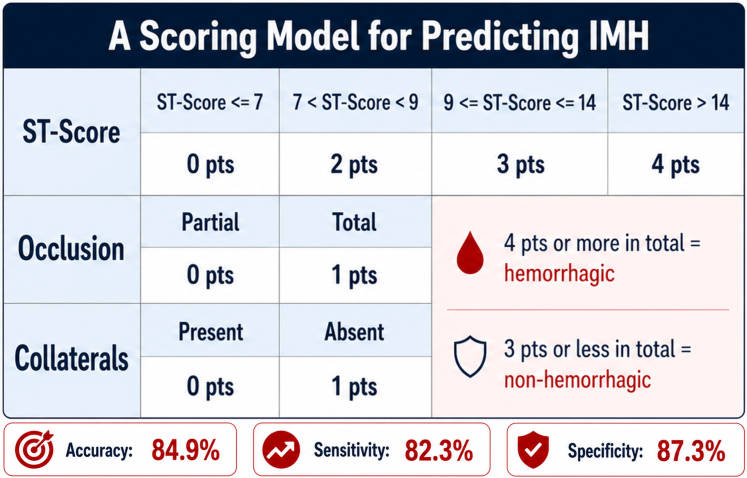
**Superposable Neural Network-Derived Bedside Intramyocardial Hemorrhage Score** Point-based bedside score derived from the final SNN for intraprocedural prediction of intramyocardial hemorrhage (IMH). Points are assigned according to summed ST-segment elevation, degree of culprit-vessel occlusion, and collateral status. The total score classifies patients as lower or higher risk for IMH, with a prespecified decision threshold of 4 or greater. This score preserves the decision logic of the additive SNN while simplifying application during emergency coronary angiography before reperfusion.

After training the SNN model, we assessed its performance using ROC analysis and selected an operating threshold of 0.63, balancing sensitivity and specificity. The learned SNN contribution functions were then inspected to identify decision regions, that is, combinations of sum ST-score, culprit-vessel occlusion status, and collateral status for which summed SNN output exceeded this threshold and indicated elevated risk of IMH. To translate the model into a practical bedside tool, these SNN-derived decision regions were expressed as an additive integer system over four ST-score categories, 2 occlusion categories, and 2 collateral categories identified from the XAI model. Reference categories were assigned 0 points, whereas total occlusion and absent collaterals were each assigned 1 point as adverse binary modifiers. Solving the resulting inequality system yielded ST-score category points of 0, 2, 3, and 4. The resulting score ranges from 0 to 6 points, with a total score ≥4 predicting IMH. The 4-point decision threshold was selected because 4/6 most closely matched the standalone high-risk SNN function value of 0.65 corresponding to rule 1. This scoring system simplifies the prediction process into an easy-to-use format, enabling clinicians to assess risk in real time. Visual illustrations of this process and the final predictive scoring model are presented in Figures 2 and 3, respectively.

### Model training

We divided the patient cohort into 2 partitions: a training set comprising 70% of the data for model development and an internal validation set comprising the remaining 30%. The performance of the SNN model was assessed using the AUC. To benchmark its predictive capability, the model's results were compared with the traditional state of the art black-box machine learning teacher models, Multi-Stage Training^25^ and Random Forest.^26^ Using 10-fold cross validation. All feature/interaction selection and model fitting steps (including teacher model training and distillation) were performed using the training partition only. Model development incorporated 10-fold cross-validation during teacher-model evaluation, followed by assessment of the locked SNN in a held-out internal validation/test partition. The separate internal test partition was used only to assess whether performance of the locked SNN was concordant with that observed during cross-validated model development. Finally, the point-based bedside score derived from the SNN was applied unchanged, using prespecified thresholds and no refitting, in an independent prospective external cohort.

### Predictive score vs intramyocardial hemorrhage volume

We analyzed data from patients identified as likely to develop IMH using our proposed predictive scoring model, to investigate the relationship between predictive score and IMH volume. Patients identified to be hemorrhagic (predictive score >3) were stratified into 3 groups based on their total predictive scores: 4, 5, and 6 points. IMH volume at each predictive score group was quantified using T2∗ CMR as outlined earlier and SE was computed to reflect within-group variability. As the prediction model was trained using a binary IMH outcome, this analysis should be considered exploratory/hypothesis-generating in evaluating whether higher predicted risk aligns with greater hemorrhage burden among CMR-confirmed IMH cases.

### Statistical analysis

The performance of the point-scoring system derived from the SNN model was evaluated using statistical metrics including accuracy, sensitivity, and specificity. Two-sided 95% CIs for model performance measures were estimated using a stratified nonparametric bootstrap. Statistical significance of AUC results was determined with z statistics of the SE obtained using DeLong’s test.^27^ Continuous variables were compared between patients with and without IMH using Student’s t-test. The composite bedside score was also evaluated as a three-tier categorical variable of low (0-2), intermediate (3-4), and high (5-6), and its association with IMH was tested using the chi-square test. To quantify the effect size of the score as a continuous predictor, a univariable logistic regression estimated the OR for IMH per 1-point increase with 95% CIs. Statistical significance was set with *P* < 0.01.

The final clinical output is a (0-6) point bedside score intended for ordinal risk stratification, not a continuous absolute probability calculator. Therefore, Calibration of the bedside point-based score was assessed by comparing observed IMH incidence across the prespecified low (0-2), intermediate (3-4), and high (5-6) strata. These cutoffs were prespecified for clinical interpretability, anchored around the binary decision threshold for the score (≥4), and were not defined by tertiles nor separately optimized to maximize discrimination. External validation performance was assessed using the same prespecified score thresholds derived in the development cohort. No post hoc recalibration or refitting was performed in the external validation cohort. All statistical analyses were performed using R (R Core Team, 2024).

## Results

### Predictor selection

Relative importance of individual predictors, as ranked by the XAI approach, is shown in (Figure 2B). The key predictors identified as the optimal predictor combination for predicting IMH were sum ST-score, degree of coronary occlusion, and presence/absence of coronary collaterals. The SNN model found that just 2 functions were sufficient for predicting IMH. The first is a function of the interaction between sum ST-score and coronary occlusion predictors (Figure 2C). The second is a function denoting the presence of collaterals (Figure 2D). Notably, interactions between predictors, such as sum of ST-segment elevation and coronary occlusion, contributed significantly to the predictive risk of IMH. Accordingly, 3 parameters, all from *cath lab* predictors were sufficient for accurate prediction of IMH. Patient clinical characteristics are presented in (Table 1).

**Table 1:** Clinical Characteristics

	IMH- (Nonhemorrhagic) (n = 146)	IMH+ (Hemorrhagic) (n = 142)	*P* Value
Admission parameters			
Age (years)	55.65 ± 9.68	56.60 ± 11.48	0.44
Male	114 (78.08%)	117 (82.39%)	0.45
Body mass index (kg/m^2^)	26.31 ± 4.41	26.04 ± 4.00	0.58
ST score (median [IQR])	6 [4-8]	13 [9-19]	<0.001
Angiographic parameters			
Culprit artery			0.29
LAD	90 (61.64%)	97 (68.31%)	
RCA	30 (20.55%)	27 (19.01%)	
LCX	20 (13.70%)	11 (7.75%)	
Lesion characteristics			
Proximal lesion	97 (66.44%)	111 (78.17%)	0.04
Total occlusion	37 (25.34%)	94 (66.20%)	<0.001
Coronary collaterals			<0.001
Absent	43 (29.45%)	101 (71.13%)	
Present	103 (70.55%)	41 (28.87%)	
Comorbidities			
Hypertension	59 (40.41%)	57 (40.14%)	0.96
Diabetes	38 (26.03%)	32 (22.54%)	0.58
Dyslipidemia	56 (38.36%)	47 (33.10%)	0.42
Smoking	68 (46.58%)	61 (42.96%)	0.6
CMR measurements			
BSA (m^2^)	1.87 ± 0.18	1.86 ± 0.24	0.46
LV mass (g)	108.15 ± 24.89	113.48 ± 26.34	0.08
Infarct size (%LV)	17.39 ± 13.42	42.47 ± 17.91	<0.001
MVO vol (%LV)	1.34 ± 2.45	10.54 ± 8.58	<0.001
IMH vol (g)	-	8.06 ± 5.37	-
IMH vol (%LV)	-	7.51 ± 5.64	-
LVEF (%)	47.01 ± 10.76	36.89 ± 7.22	<0.001
RVEF (%)	55.40 ± 8.95	57.93 ± 7.10	0.01

Clinical characteristics of the 288 patients involved in the study (252 were used for model development and 36 were used for validation), all of whom presented with ST-segment elevation myocardial infarction and underwent guideline-directed primary percutaneous coronary intervention. Eligibility required complete pre-procedure clinical data and post-procedure data inclusive of T2∗ CMR imaging (CMR) to assess for intramyocardial hemorrhage. Shown are both *cath lab* (electrocardiographic and angiographic features) and *clinical* risk factors (demographics and comorbidities) collected at the time of emergency coronary angiography. IMH + denotes STEMIs that were hemorrhagic and IMH- are STEMIs that are non-hemorrhagic.

BSA = body surface area; CMR = cardiac magnetic resonance; IMH = intramyocardial hemorrhage; LAD = left anterior descending; LCx = left circumflex; LV = left ventricular; LVEF = left ventricular ejection fraction; MVO = microvascular obstruction; RCA = right coronary artery; RVEF = right ventricular ejection fraction; vol = volume.

### Statistical analysis

The SNN model exhibited strong predictive performance in identifying IMH, achieving an AUC of 0.92 in the held-out validation set (Figure 2E), consistent with the 10-fold cross-validated performance of the multistage training teacher model (AUC: 0.92 ± 0.03). The composite score was higher in patients with IMH than in those without (4.38 ± 1.48 vs 1.48 ± 1.41; *P* < 0.001). Using the prespecified three-tier categorization of low (0-2), intermediate (3-4), and high (5-6), IMH occurred in (13.5%), (53.8%), and (98.6%) patients, respectively (chi-square = 128.58; *P* < 0.001), supporting risk-stratified calibration of the bedside score. In univariable logistic regression, each 1-point increase in composite score was associated with higher odds of IMH (OR: 2.90; 95% CI: 2.34-3.72; *P* < 0.001).

### Point-based predictive scoring model

The rules derived from the SNN model for prediction of IMH were.

• Rule 1: Sum ST-score >14
• Rule 2: Sum ST-score ≥9 and either total occlusion or absent collaterals
• Rule 3: Sum ST-score >7 with total occlusion and absent collaterals

The point-based predictive scoring model achieved an accuracy of 84.9% (95% CI: 80.30% -89.02%), sensitivity of 82.3% (95% CI: 75.38%-88.46%), and specificity of 87.3% (95% CI: 81.34%-92.54%). In the external validation cohort (n = 36), the finalized point-based score achieved similar metrics: accuracy of 83.3%; sensitivity of 81.3%; and specificity of 85%. Figure 3 illustrates the specified number of points assigned to each predictor based on its value, where the total number of points determines whether the patient will be hemorrhagic when it exceeds the specified threshold. For example, a patient with no coronary collaterals, a partial occlusion, and sum ST-score of 10 has a *predictive score* of 4 (1 + 0 + 3), thus expected to develop IMH postreperfusion. In another example, a patient with coronary collaterals, also with a partial occlusion, and a sum ST-score of 12 has *predictive score* of 3 (0 + 0 + 3) and will not be expected to develop IMH.

### Representative examples

**Figure 4:**
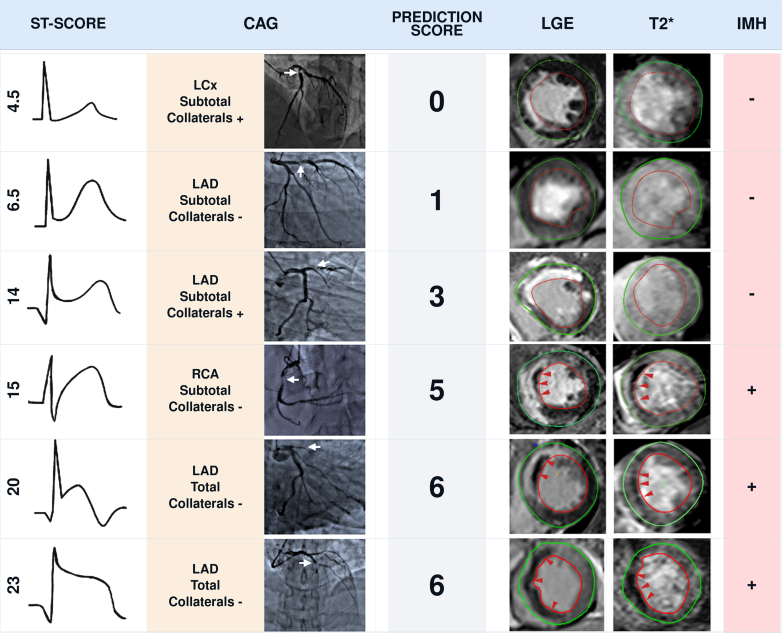
**Prospective Model Validation: Intramyocardial Hemorrhage and Non-Intramyocardial Hemorrhage Cases** Representative non-IMH and IMH cases from the study. The first column shows pre-PCI ECG examples and summed ST-segment elevation. The second column shows coronary angiography, including degree of occlusion and collateral status. The third column shows the bedside score, with scores < 4 classified as non-IMH and scores 4 or greater classified as IMH. The fourth and fifth columns show CMR confirmation of infarction and intramyocardial hemorrhage using late gadolinium enhancement and T2∗ imaging, respectively. Red contours mark the endocardium, green contours mark the epicardium, and arrows indicate microvascular obstruction or IMH where present. CAG = coronary angiography; IMH = intramyocardial hemorrhage; LGE = late gadolinium enhancement.

To validate the performance of the SNN model in predicting IMH in real-world scenarios, 6 prospective patients were analyzed. Three nonhemorrhagic and 3 hemorrhagic patient examples are presented in Figure 4. These cases illustrate how the model's rules were applied based on clinical data from STEMI patients undergoing PCI.

#### Case 1

A 57-year-old male presented with a sum ST-score of 4.5. Coronary angiography indicated subtotal occlusion of the LCX with coronary collaterals present. The SNN did not predict IMH as the sum ST-score contributed 0 points, subtotal occlusion contributed 0 points, and the presence of collaterals contributed 0 points, resulting in a total *predictive score* of *0 points*, classifying the patient as *nonhemorrhagic*. The absence of IMH was confirmed by T2∗ CMR performed 48 hours post-PCI.

#### Case 2

A 62-year-old female presented with anterior STEMI and a sum ST-score of 6.5. Coronary angiography revealed subtotal occlusion of the LAD artery, with absence of collaterals. This patient also did not meet the model’s prediction threshold for IMH: the sum ST-score contributed 0 points, subtotal occlusion contributed 0 points, and the absence of collaterals contributed 1 point, resulting in a total *predictive score* of 1 point, suggesting that the patient will be nonhemorrhagic. The patient was confirmed to be nonhemorrhagic based on T2∗ CMR performed 48 hours post-PCI.

#### Case 3

A 62-year-old male presented with anterior STEMI and a sum ST-score of 14. Coronary angiography revealed subtotal occlusion of the LAD artery, with presence of collaterals. This patient also did not meet the SNN model's threshold for predicting IMH. Based on the point scoring system, the sum ST-score contributed 3 points, subtotal occlusion contributed 0 points, and the presence of collaterals contributed 0 points, resulting in a total *predictive score* of 3 points. Hence, the model predicted the patient will be nonhemorrhagic in the peri-PCI period. T2∗ CMR performed 48 hours post-PCI confirmed that the patient was nonhemorrhagic.

#### Case 4

A 52-year-old male presented with inferior STEMI and a sum ST-score of 15. Coronary angiography revealed subtotal occlusion of the RCA, with absence of collaterals. This patient met the criteria as per rule 1 (sum ST-score >14) and rule 2 (sum ST-score >9 and absent collaterals), exceeding the SNN model's threshold for predicting IMH. Based on the point scoring system, the sum ST-score contributed 4 points, subtotal occlusion contributed 0 points, and the absence of collaterals contributed 1 point, for a total *predictive score* of 5 points, which predicted the patient will be hemorrhagic, and was confirmed to be the case by T2∗ CMR 48 hours post-PCI.

#### Case 5

A 53-year-old male presented with anterior STEMI and a sum ST-score of 20. Coronary angiography revealed total occlusion of the LAD artery, with the absence of collaterals. Based on the point scoring system, the sum ST-score contributed 4 points, total occlusion contributed 1 point, and the absence of collaterals contributed 1 point, resulting in a total *predictive score* of 6 points, which predicted that the patient will be hemorrhagic. T2∗ CMR performed 48 hours post-PCI indeed confirmed the patient to be hemorrhagic.

#### Case 6

A 37-year-old male presented with anterior STEMI and a sum ST-score of 23. Coronary angiography revealed total occlusion in the LAD artery, with absence of collaterals. Based on the point scoring system, the sum ST-score contributed 4 points, total occlusion contributed 1 point, and the absence of collaterals contributed 1 point, resulting in a total *predictive score* of 6 points, which predicted that the patient will be hemorrhagic. T2∗ CMR performed 48 hours post-PCI confirmed the patient to be hemorrhagic.

### Relation between predictive score and intramyocardial hemorrhage volume

**Figure 5:**
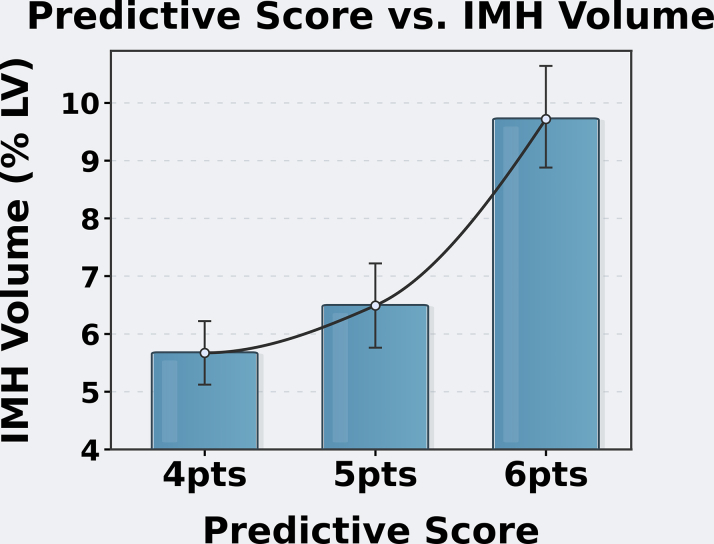
**Relationship Between Predictive Scores and Intramyocardial Hemorrhage Volume** This figure illustrates the mean intramyocardial hemorrhage (IMH) volume, along with standard error, from patients with hemorrhagic MI stratified by Predictive Score groups from our proposed model. Patients with a Predictive Score of 4 or higher developed IMH with volume as %LV determined using T2∗ CMR. A clear positive relationship was observed, where higher predictive scores were associated with greater IMH volume. LV = left ventricular.

The relationship between predictive score and IMH volume is illustrated in Figure 5. Patients with a predictive score of 4 exhibited a mean IMH volume of approximately 5.7% of the LV volume, whereas those with scores of 5 and 6 demonstrated mean volumes of 6.5% LV and 9.8% LV, respectively. A clear positive relationship was observed, wherein higher predictive scores were associated with increased IMH volume. The observed trend indicates that patients with elevated risk scores, as defined by our proposed predictive scoring model, are more likely to develop more extensive myocardial hemorrhage. These findings support the model’s utility in identifying patients at greater risk for extensive hemorrhagic severity following MI.

**Central Illustration:**
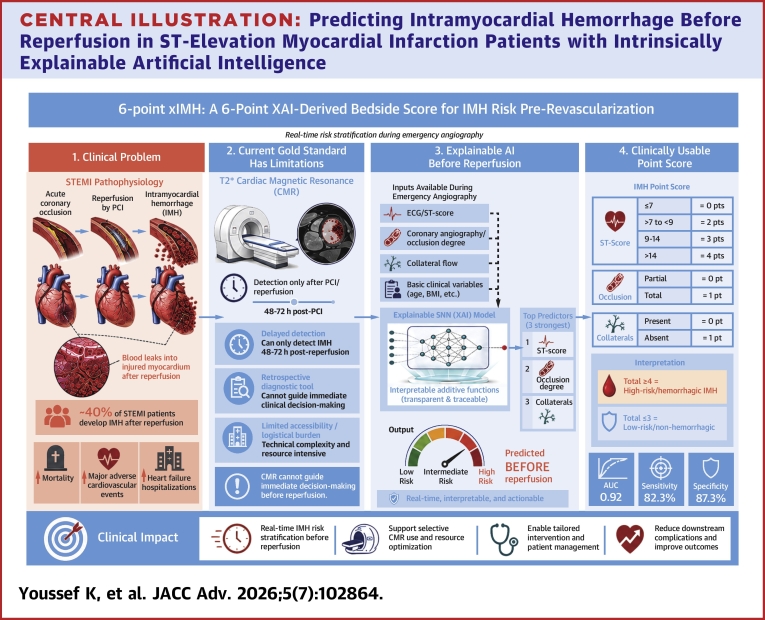
**Predicting Intramyocardial Hemorrhage Before Reperfusion in ST-Elevation Myocardial Infarction Patients with Intrinsically Explainable Artificial Intelligence** ECG = electrocardiogram; PCI = percutaneous coronary intervention; SNN = Superposable Neural Network; STEMI = ST-segment elevation myocardial infarction; XAI = explainable artificial intelligence.

## Discussion

IMH following mechanical revascularization is a major clinical complication in STEMI patients and contributes to a multifold greater risk for MACE, including heart failure and death. Although CMR has emerged as a robust method for identifying hemorrhagic MI patients, determination of whether a STEMI patient is likely to experience IMH prior to revascularization is currently not possible. If an approach were to be available for identifying the vulnerable patient, it would offer open opportunities to optimize interventions to minimize or prevent the development of IMH. Guided by this need, we investigated whether an XAI-based algorithm can yield a predictive scoring system that has the capability to be deployed in real-time in busy interventional settings to identify patients at risk of developing IMH. We used all the clinical data available to the interventional cardiology team at the time of emergency PCI in STEMI patients, including ECG, angiographic parameters (comprising degree of coronary occlusion, evidence of coronary collaterals, culprit artery, and lesion location), along with clinical comorbidities to identify the predictors of IMH. Our findings showed that a rapidly deployable predictive scoring system, which is grounded in XAI, can identify patients at risk of developing IMH based on ECG and angiographic parameters readily accessible in the interventional setting. Unlike traditional "black-box" predictive models whose internal workings remain opaque, our XAI-based framework provides transparency and interpretability, ensuring that predictions can be understood and trusted by clinicians. Although we present a point-based score for bedside usability, interpretability is already intrinsic at the model level because the SNN prediction is an explicit sum of independent feature (and composite-feature) contributions. This represents a fundamental shift in how predictive artificial intelligence can be used in cardiology, offering not only powerful prognostic capabilities but also actionable insights that can inform real-time clinical decision-making.

An important insight from our study is the importance of *cath lab* variables that evolve during the "pain-to-balloon" period, including the degree of coronary occlusion, ST-segment elevation score, and presence of collaterals, as the strongest predictors of IMH. These variables, reflective of real-time myocardial injury, offer more immediate and actionable information than standard *clinical* parameters. The predictive value of these *cath lab* predictors aligns with existing clinical understanding, reinforcing the importance of assessing the dynamic physiological state of patients during STEMI. Furthermore, our findings also revealed that the predictive scores are directly related to the extent of IMH volume. This provides additional insight into the extent of myocardial injury the patient to be revascularized is at risk of developing after revascularization, given that the extent of hemorrhage volume post-PCI and infarct size are directly related.^3^ It may seem surprising that traditional baseline risk factors (eg, age, diabetes, and hypertension) were not retained as primary predictors. In our cohort, these variables were included among candidate predictors but did not provide incremental predictive value once acute pathophysiology-proximal markers of ischemic severity and microvascular compromise available at the time of angiography (summed ST-score, degree of occlusion, and collateral status) were incorporated. This pattern is biologically plausible as IMH is an acute reperfusion-injury phenotype largely driven by the severity of the culprit-lesion physiology and downstream microvascular disruption, which these cath lab markers capture more directly than chronic comorbidity profiles. Nevertheless, we cannot exclude smaller contributions of traditional risk factors that may require larger and more heterogeneous cohorts to detect reliably. Furthermore, although the extracted decision rules appear clinically intuitive, it is important to emphasize that they were not manually engineered; rather, they are a discretized bedside translation of the continuous additive decision function learned by the SNN. The incremental value of the SNN framework is that it: 1) systematically evaluates candidate predictors and composite-feature interactions; 2) learns the *shape* of each predictor’s contribution as a nonlinear function while maintaining full transparency; and 3) compresses high-capacity teacher behavior into a compact, intrinsically explainable additive model with an exact decomposition of risk into per-feature (and per-interaction) terms. In principle, similar threshold-style rules might be approximated with a decision tree or a logistic regression could be constructed with carefully chosen splines and interaction terms, but these approaches generally require greater manual specification and/or may yield less stable, less portable structures when the goal is to capture nonlinear effects with minimal complexity.

### Study limitations

As this is the first study to demonstrate the feasibility of the approach, additional cohorts are needed to ensure the generalizability of our findings to broader and more diverse populations. Given the modest sample size of the external validation cohort, further validation in larger, multicenter cohorts is necessary to confirm the robustness of the model across different clinical settings. Second, although the point-based predictive scoring model provides a practical approach to real-time prediction, additional prospective studies are needed to evaluate its utility and benefits in clinical decision-making and patient outcomes in real-world settings. Third, although investigations into IMH are limited to STEMI population, whether non-STEMI patients can also experience IMH is not known and was not assessed in this study. In addition, we also recognize that the modest sample size may limit power to detect small incremental effects of traditional risk factors (eg, age, diabetes, and hypertension) beyond the dominant acute angiographic/ECG predictors.

## Conclusions

We conclude that our findings advance the prediction of IMH before revascularization based on an XAI algorithm, which offers both a high predictive accuracy and interpretability. Future work should explore the model's applicability in larger patient populations and investigate its role in enhancing clinical workflows, with the goal of improving patient care and outcomes.

Perspectives **COMPETENCY IN MEDICAL KNOWLEDGE:** IMH following reperfusion in STEMI has emerged as the key driver of post-MI complications. As only 40% of STEMI patients develop IMH, methods that can identify which STEMI patients are at the greatest risk for IMH could be a major advance improving STEMI care.

**TRANSLATIONAL OUTLOOK:** Clinical parameters accessible in the cardiac catheterization labs immediately prior to reperfusion, when combined with explainable AI, can identify those at risk of developing of IMH. This knowledge can help unravel strategies to mitigate/prevent the development of IMH before it happens, identify patients who may benefit from novel pharmacotherapies targeting IMH, and for patient selection for clinical trials that can benefit from understanding the role of IMH in STEMI patients.

## Funding support and author disclosures

This work was funded in part by 10.13039/100000002NIH/10.13039/100000050NHLBI (HL133407, HL136578 and HL147133) to Dr Dharmakumar. Dr Dharmakumar has equity interest in Cardio-theranostics LLC. All other authors have reported that they have no relationships relevant to the contents of this paper to disclose.
